# Clinical Utility of the 6-Item CTS, Boston-CTS, and Hand-Diagram for Carpal Tunnel Syndrome

**DOI:** 10.3389/fneur.2021.683807

**Published:** 2021-07-27

**Authors:** Daniel Gregor Schulze, Kristian Bernhard Nilsen, Rikke Munk Killingmo, John Anker Zwart, Margreth Grotle

**Affiliations:** ^1^Department of Neurology, Oslo University Hospital and University of Oslo, Oslo, Norway; ^2^Institute of Clinical Medicine, University of Oslo, Oslo, Norway; ^3^Department of Research, Innovation and Education, Division of Clinical Neuroscience, Oslo University Hospital, Oslo, Norway; ^4^Oslo Metropolitan University, Oslo, Norway

**Keywords:** clinical utility, Norwegian, 6-item CTS, hand-diagram, carpal tunnel syndrome, Boston CTS questionnaire, COSMIN checklist

## Abstract

**Background:** Self-reported measures are often used in research and clinical practice to diagnose carpal tunnel syndrome (CTS) and guide therapeutic choices. We aimed to assess the clinical utility of the Norwegian versions of two self-reported outcome measures for symptom severity assessment, the 6-item CTS (CTS-6), and Boston-CTS (BCTQ), and of one diagnostic measure, the hand-diagram, by evaluating measurement properties including discriminative ability for severity assessment (CTS-6, BCTQ), and diagnosis of CTS (hand-diagram).

**Methods:** We performed forward and backward translation and cultural adaptation of the Norwegian CTS-6 and BCTQ. Following COSMIN guidelines, we investigated internal consistency, reliability, construct validity, and discriminative ability for distinguishing between severity levels of CTS in patients with confirmed CTS for the CTS-6 and BCTQ and reliability and discriminative ability for diagnosing CTS for the hand-diagram.

**Results:** Two hundred and fifty-one patients referred for diagnostic work-up for CTS with nerve conduction studies (NCS) participated. The CTS-6 and BCTQ had acceptable internal consistency (Crohnbach's α = 0.82 and 0.86, respectively), reliability (ICC = 0.86 and 0.90; SEM = 0.24 and 0.20; SDC95% = 0.68 and 0.55, respectively), construct validity (all eight pre-defined hypotheses confirmed) and discriminative ability to distinguish between severity levels of CTS [Area under the curve (AUC) = 0.75, 95% CI 0.64–0.85]. The hand-diagram had acceptable reliability (Cohen's kappa = 0.69) and discriminative ability to diagnose CTS (sensitivity = 0.72, specificity = 0.90).

**Conclusion:** Our findings support the clinical utility of the CTS-6 and BCTQ for symptom severity assessment and of the hand-diagram for diagnostic screening.

## Introduction

Carpal tunnel syndrome (CTS) is characterized by paresthesia, numbness and pain in the median nerve distribution (1). Nerve conduction studies (NCS) contribute to diagnosing CTS by demonstrating reduced conduction velocity of the median nerve (2). Treatment is often based on clinical and NCS severity (3, 4). Due to a CTS prevalence of ca. 3–5% (5) and limited access to NCS, several measures (6, 7) have been developed for diagnostic screening and for severity assessment. The 6-item CTS from Atroshi et al. (CTS-6) (8, 9) and Boston carpal tunnel questionnaire (BCTQ) (10) are widely used for severity assessment, and the hand-diagram (11, 12) for diagnostic screening. These instruments are not currently available in Norwegian. Single measurement properties have been described for other language versions of these instruments (13, 14), but few studies follow systematic guidelines, as for instance provided by the COSMIN group (15). Thus, it can be challenging to choose from among the available instruments (16, 17) and measures may not be used as designed or for their intended purpose, greatly hampering their utility. An example is that several studies used the BCTQ for diagnostic purposes (18). Thus, a study of the measurement properties of these instruments according to the COSMIN guidelines, together with a delineation of their respective discriminative abilities for diagnosis (hand-diagram) and severity assessment [CTS-6 and (BCTQ)], could give a good estimate of their utility and help clinicians and researchers to choose the appropriate instrument (19).

The main objective of this study was to systematically investigate internal consistency, reliability, construct validity and discriminative ability for severity assessment of the Norwegian versions of the CTS-6, BCTQ, and reliability and discriminative ability of the hand-diagram for diagnosing CTS according to the COSMIN guidelines (20).

## Methods

### Design

The study was carried out in two stages. First, we translated and cross-culturally adapted the CTS-6 and BCTQ. Then, we used a cross-sectional design to test the measurement properties of the CTS-6, BCTQ, and hand-diagram against NCS as external criteria. Additionally, we performed a test–retest assessment with an interval of 4 days.

Following COSMIN recommendations (21), we aimed for a sample size of 200 patients, consisting of 100 patients with confirmed CTS and 100 patients for whom CTS could not be confirmed. Internal consistency and the discriminative ability to distinguish severity levels of CTS (both analyses applied to the CTS-6 and BCTQ) was tested in the sample with confirmed CTS, while construct validity (applied to the CTS-6 and BCTQ) and discriminative ability for diagnosis of CTS (applied to the hand-diagram) were tested in the entire sample. For test–retest reliability, we invited the first fifty included patients with confirmed CTS to complete a retest questionnaire 4 days after the baseline questionnaire. According to Norwegian law, this study was categorized as a quality improvement project and not a medical research project. The Study was accordingly approved by the local Data Protection Official (PVO 2015/14753) and not the Regional Ethical Committee. All participants provided written consent.

### Participants

We recruited patients who had been referred for diagnostic work-up of suspected CTS with NCS to the clinical neurophysiology lab at Oslo University Hospital. We included patients referred from both primary and secondary health services. Patients > 18 years of age with sufficient knowledge of the Norwegian language were eligible. Exclusion criteria were patient withdrawal or more than 50% missing items in the questionnaire. Written informed consent was obtained from all patients.

### Procedures and Measures

The participants filled out the CTS-6, BCTQ, hand-diagram, and sociodemographic background variables 2 days before the consultation. Those participating in test–retest reliability assessment were asked to fill out the CTS-6, BCTQ, and hand-diagram again 2 days after the consultation and return them by mail. We chose an interval of 4 days in order to minimize bias from memory or from a change in clinical symptoms. For each questionnaire, patients were asked to state for which hand they had answered.

### Translation and Cross-Cultural Adaptation

The translation and cross-cultural adaptation was conducted according to international guidelines (22), including forward translation performed by a native speaker of Norwegian and backward translations performed by native speakers of English and Swedish. Based on the translations, an expert committee developed a pre-final version. Following a review by 10 patients with musculoskeletal disorders recruited from our out-patient clinic, a final version was developed. The Norwegian versions of the CTS-6 and BCTQ are provided in Supplementary Materials 1, 2.

### Measures

The CTS-6 (8) was developed from the BCTQ as a brief symptom scale for patients with confirmed CTS. It was designed to measure severity of the cardinal symptoms of CTS. The questionnaire consists of six items covering presence and intensity of numbness, paresthesia, and pain during day and night, whether the patient is woken by these symptoms, and, if so, how often. Each question is answered on a scale ranging from “1” (best) to “5” (worst). The total score is calculated as the mean of all answers, with a minimum score of “1” and a maximum score of “5.” One missing item is allowed.

The BCTQ (10) was designed as a self-reported measure of symptom severity and functional impairment of CTS, with two respective subscales. Only the symptom severity scale was used in the present study. This scale consists of 11 items covering presence, frequency, duration and severity of numbness, paresthesia and pain, both day and night, and impairment of fine motor skills, with five possible answers ranging from “1” (best) to “5” (worst). The total score is calculated as the mean of all answers, with a minimum score of “1” and a maximum score of “5.” One missing item is allowed.

The hand-diagram (11) was developed as a rapid screening tool for CTS in the general population. It consists of a diagram of the palmar and dorsal hand, and patients record in which areas of the hand and arm they experience numbness, tingling, pain and loss of sensation. There are four different probability scores according to presence and distribution of symptoms: (1) Classic CTS is defined by tingling, numbness, decreased sensation with or without pain in at least two of the three radial digits. Symptoms in the palm and dorsum of the hand are not allowed, wrist pain or radiation proximal to the wrist is allowed. (2) Probable CTS is defined by the same pattern as classic CTS, except that palmar symptoms are allowed unless confined to the ulnar aspect. (3) Possible CTS is defined by tingling, numbness, decreased sensation, and/or pain in at least one of the three radial digits. (4) Unlikely CTS is defined by the absence of symptoms in the three radial digits.

### Nerve Conduction Studies

We performed bilateral motor and sensory NCS of the median and ulnar nerves on a Natus key-point classic EMG machine (Alpine Bio-med, Denmark). For both motor and sensory NCS, we used pre-gelled, disposable surface electrodes (Alpine biomed, Skovlunde, Denmark) with a 3 mm inter-electrode distance. For stimulation we used a handheld stimulation bar with felt tips (diameter 7.5 mm) soaked in saline solution and with fixed inter-electrode distance. Electrodes were placed on predefined anatomic landmarks and distances between stimulation and recording sites were measured prior to performing NCS. Before conducting the NCS, we measured skin temperature with a handheld infrared thermographic scanner (Exergen Corporation, Watertown, MA, USA). Skin temperature was kept over 30 degrees at all times. All amplitudes were recorded using supramaximal stimulation. Motor and sensory amplitudes were measured from baseline to peak. Sensory latencies were calculated based on the peak of the negative deflection, motor latencies at onset of the negative peak. We performed orthodromic sensory NCS of the median nerve (branches to the palm and to the second, third, and fourth fingers), ulnar nerve (branches to the palm and to the fourth and fifth fingers) and radial nerve (superficial branch at the laterodorsal side of the hand). Distances between stimulation and recording electrodes in the fourth finger were equal in the median and ulnar nerves to allow for comparison of the sensory latency. NCS findings were classified according to the scale suggested by Padua (23). Minimal CTS is characterized by a significant difference between sensory latency in the median/ulnar nerves at two sites (≥0.5 ms in the fourth digit and in the palm); mild CTS by sensory conduction velocities of the median nerve below the lower normal limit; moderate CTS by motor distal latency above the normal limit in addition to sensory involvement; severe CTS by additionally absent sensory responses (amplitude <0.2 mV); and extreme CTS by absence of both motor and sensory responses.

### Diagnosing CTS

In order to provide a CTS diagnosis certain clinical (1, 2) and NCS criteria had to be met: A diagnosis of CTS was present if NCS showed at least minimal CTS according to Padua (23) and two of the following classical symptoms of CTS (2) were present: numbness and/or paresthesia in the median nerve distribution, alleviation of symptoms by shaking the limb, weakness in the hand and presence of symptoms during the night. Further, there should be no alternative or more plausible explanation for these symptoms. In cases of bilateral disease, analyses were applied to the most symptomatic side.

### Statistical Analysis

The analysis was performed on SPSS V24 software (SPSS Inc., Chicago, IL). *P* < 0.05 were considered significant. We substituted up to 50% missing items with the mean of the remaining answers. Floor and ceiling effects were defined as >15% of patients reporting the lowest or highest possible total score, and end-effects as >15% of patients reporting the highest or lowest possible score for a single item.

#### Internal Consistency

Internal consistency was assessed by Cronbach's α. COSMIN regards values of >0.70 and <0.95 as acceptable.

#### Test–Retest Reliability

Test–retest reliability was tested with relative and absolute reliability measures. Relative reliability was assessed with intra-class correlation (ICC_2.1_, two way random, absolute agreement) (24), with values of ≥0.70 regarded as acceptable. Absolute reliability was tested with the standard error of measurement (SEM, calculated as standard deviation of the difference/√2) (24), the smallest detectable change [SDC 95%, calculated as SEM × √2 × 1.96 (24)] and the limits of agreement (calculated as mean difference ± standard deviation of the difference × 1.96) (25). For ordinal variables (the hand-diagram), Cohen's quadratic weighted kappa was used (26). Kappa values were categorized as follows (21, 27): poor (0–0.20), fair (0.21–0.40), moderate (0.41–0.60), good (0.61–0.80), and very good (0.81–1.00).

#### Construct Validity

Construct validity was assessed for the CTS-6 and BCTQ by testing eight pre-defined hypotheses concerning the CTS-6, the BCTQ, and concurrent measurements based on existing literature (2, 8, 10, 11, 28–32). Hypotheses one, three, four, five, six, and seven were tested in the sample with confirmed CTS, and hypotheses two and eight in the whole sample. Since the CTS-6 and BCTQ measure the same construct, parallel hypotheses were created.

#### Discriminative Ability

Discriminative ability of the CTS-6 and BCTQ was assessed by receiver operating characteristics (ROC) curves for the ability to distinguish between minimal to mild and moderate to severe CTS grades. An area under the curve (AUC) of at least 0.70 is considered adequate (21). We used the coordinates of the ROC curve to calculate the scores with optimal sensitivity and specificity and positive and negative likelihood ratios. We assessed the discriminative ability of the hand-diagram by calculating sensitivity, specificity, and positive and negative likelihood ratios directly from 2 × 2 tables for the diagnostic ability to discriminate between patients who had or had not been diagnosed with CTS.

## Results

We collected data from April 2016 to January 2018. Out of 293 invited patients, 42 either declined to participate or did not speak Norwegian. Of the 251 remaining patients, 128 were diagnosed with CTS (based on predefined clinical symptoms and NCS findings). Of the 123 patients who were not diagnosed with CTS, 10 did not fulfill the clinical criteria, 15 did not fulfill the NCS criteria and 98 did not fulfill either of these criteria. These patients were diagnosed with one of the following conditions: polyneuropathy, ulnar nerve neuropathy, cervical disk herniation, tendinitis, or radial nerve neuropathy. Internal consistency and the discriminative ability to distinguish between severity levels of CTS (applied to the CTS-6 and BCTQ) was tested in the sample with confirmed CTS, while construct validity and discriminative ability for diagnosis of CTS (applied to the hand-diagram) were tested in the entire sample. Fifty-four patients with confirmed CTS participated in test–retest analysis. Sample characteristics are given in Table 1. There were no significant differences between the samples regarding age, sex distribution, educational level, or proportion of patients with Norwegian as their mother tongue.

**Table 1:** Sample characteristics of the whole sample (both confirmed and non-confirmed carpal tunnel syndrome [CTS]), of the sample with confirmed CTS alone and of the test–retest sample.

	**Whole sample**	**Confirmed CTS**	**Test–retest sample**
*N*	251	128	54
**Patient characteristics**			
Female, *N* (%)	167 (66.5)	85 (66.4)	35 (64.8)
Age in years, mean(SD)	51.4 (15.0)	54 (15.0)	57 (16.0)
Smoking status (% yes)	41 (16.3)	21 (16.4)	4 (7.4)
**Educational level**, ***N*****(%)**			
Primary/lower secondary school	33 (13.1)	15 (11.7)	5 (9.3)
Upper secondary school	81 (32.3)	41 (32.1)	15 (27.7)
College or university	117 (46.6)	66 (51.6)	32 (59.3)
Missing	20 (8.0)	6 (4.6)	2 (3.7)
Mother tongue Norwegian, *N* (%)	170 (67.7)	100 (80.6)	45 (83.3)
**Symptom duration**, ***N*****(%)**			
≤3 months	19 (7.6)	11 (8.6)	4 (7.4)
3–12 months	92 (36.7)	56 (43.8)	25 (46.3)
1–2 years	34 (13.5)	12 (9.4)	3 (5.6)
≥2 years	73 (29.1)	34 (26.6)	13 (24.1)
Missing	33 (13.1)	15 (11.6)	9 (16.6)
**Nerve conduction Studies severity**, ***N*****(%)**			
Grade 0	110 (43.8)	0 (0)	0 (0)
Grade 1	10 (4.0)	7 (5.5)	2 (3.7)
Grade 2	23 (9.1)	20 (15.6)	8 (14.8)
Grade 3	73 (29.0)	69 (53.9)	34 (63.0)
Grade 4	31 (12.3)	30 (23.4)	10 (18.5)
Grade 5	4 (1.6)	2 (1.6)	0 (0)
**Hand-diagram scores**, ***N*****(%)**			
Unlikely	40 (15.9)	2 (1.5)	1 (1.8)
Possible	102 (40.6)	33 (25.7)	29 (53.7)
Probable	59 (23.5)	57 (44.5)	15 (27.8)
Classic	40 (15.9)	33 (25.7)	6 (11.1)
Missing	10 (3.9)	3 (2.3)	3 (5.5)

### Data Quality

The total scores were normally distributed in the CTS-6 and BCTQ. There was little missing data for the CTS-6 and BCTQ (Tables 2A,B). Neither of the outcome measures showed floor or ceiling effects, but there were end effects in four items of the CTS-6 and in eight of the 11 items in the BCTQ (defined as >15% of patients reporting the lowest or highest possible score for a single item). Ten patients had missing data in the hand-diagram. The distribution of hand-diagram scores is presented in Table 1.

**Table 2A:** Missing data, data distribution, end effects and total item correlation of the 6-item CTS.

	**Total score**	**Item #**	**1**	**2**	**3**	**4**	**5**	**6**
Range	1–5		1–5	1–5	1–5	1–5	1–5	1–5
Mean (SD)	2.8 (0.7)		2.7 (1.2)	2.5 (1.0)	3.4 (1.0)	3.3 (0.9)	2.3 (1.1)	2.8 (0.9)
Crohnbach's α if deleted			0.7	0.8	0.8	0.8	0.7	0.7
Proportions of lowest score	10.5%		11.1%	1.7%	18.9%	10.5%	8.0%	8.7%
Proportions of highest score	7.3%		20.5%	22.9%	4.1%	3.2%	32.7%	9.5%
Missing items *N* (%)			11 (8.5)	10 (7.8)	6 (4.6)	4 (3.1)	15 (11.7)	2 (1.5)
75th percentile score	3.3		4.0	3.0	4.0	4.0	3.0	3.0

**Table 2B:** Missing data, data distribution, end effects and total item correlation of the Boston Carpal Tunnel Questionnaire (BCTQ).

	**Total score**	**Item #**	**(1)**	**(2)**	**3)**	**(4)**	**(5)**	**(6)**	**(7)**	**(8)**	**(9)**	**(10)**	**(11)**
Range	1–5		1–5	1–5	1–5	1–5	1–5	1–5	1–5	1–5	1–5	1–5	1–5
Mean (SD)	2.8 (0.7)		2.5 (1.1)	2.6 (1.2)	2.5 (1.0)	2.8 (1.4)	2.7 (1.4)	3.5 (1.0)	2.6 (1.2)	3.0 (1.1)	3.4 (1.1)	2.9 (1.1)	2.3 (1.2)
Crohnbach's α if deleted			0.8	0.8	0.8	0.8	0.8	0.8	0.8	0.8	0.8	0.8	0.8
Proportions of lowest score	13.4%		4.8%	12%	1.6%	16.8%	23.4%	14.2%	8.7%	9.5%	20.5%	12.9%	31.7%
Proportions of highest score	7.1 %		23.8%	24.8%	23.2%	26.4%	25.8%	3.9%	23.6%	10.3%	5.5%	11.3%	7.1%
Missing items *N* (%)			2 (1.6)	3 (2.3)	3 (2.3)	3 (2.3)	4 (3.1)	1 (0.8)	1 (0.8)	2 (1.6)	1 (0.8)	4 (3.1)	2 (1.6)
75th percentile score	3.3		3.0	3.0	3.0	4.0	4.0	4.0	4.0	4.0	4.0	3.0	3.0

### Internal Consistency and Test–Retest Reliability

Cronbach's α was 0.82 for the CTS-6 and 0.86 for the BCTQ. There was no difference between the test and the re-test total scores for the CTS-6 and BCTQ (Table 3). Both the SEM and the SDC were lower for the BCTQ than for the CTS-6. The agreement and test–retest reliability were acceptable for all three instruments.

**Table 3:** Test–retest reliability and agreement for the 6-item CTS (CTS-6), the Boston Carpal Tunnel Questionnaire (BCTQ), and the hand-diagram.

	**CTS-6**	**BCTQ**	**Hand-diagram**
Range	1–5	1–5	NA
Mean (SD)	2.88 (0.77)	2.83 (0.78)	NA
Mean (SD) re-test	2.72 (0.71)	2.65 (0.65)	NA
SEM	0.24	0.20	NA
SDC 95%	0.68	0.55	NA
ICC [95% CI]	0.86 [0.78–0.92]	0.90 [0.83–0.94]	NA
Lower limit of agreement	−0.64	−0.47	NA
Upper limit of agreement	−0.72	−0.61	NA
Kappa W [95% CI]	NA	NA	0.69 [0.46–0.91]

*NA, not assessed; SD, standard deviation; SEM, standard error of the measurement; SDC, smallest detectable change; ICC, intra class correlation coefficient; kappa W, weighted kappa*.

### Construct Validity

**Figure 1:**
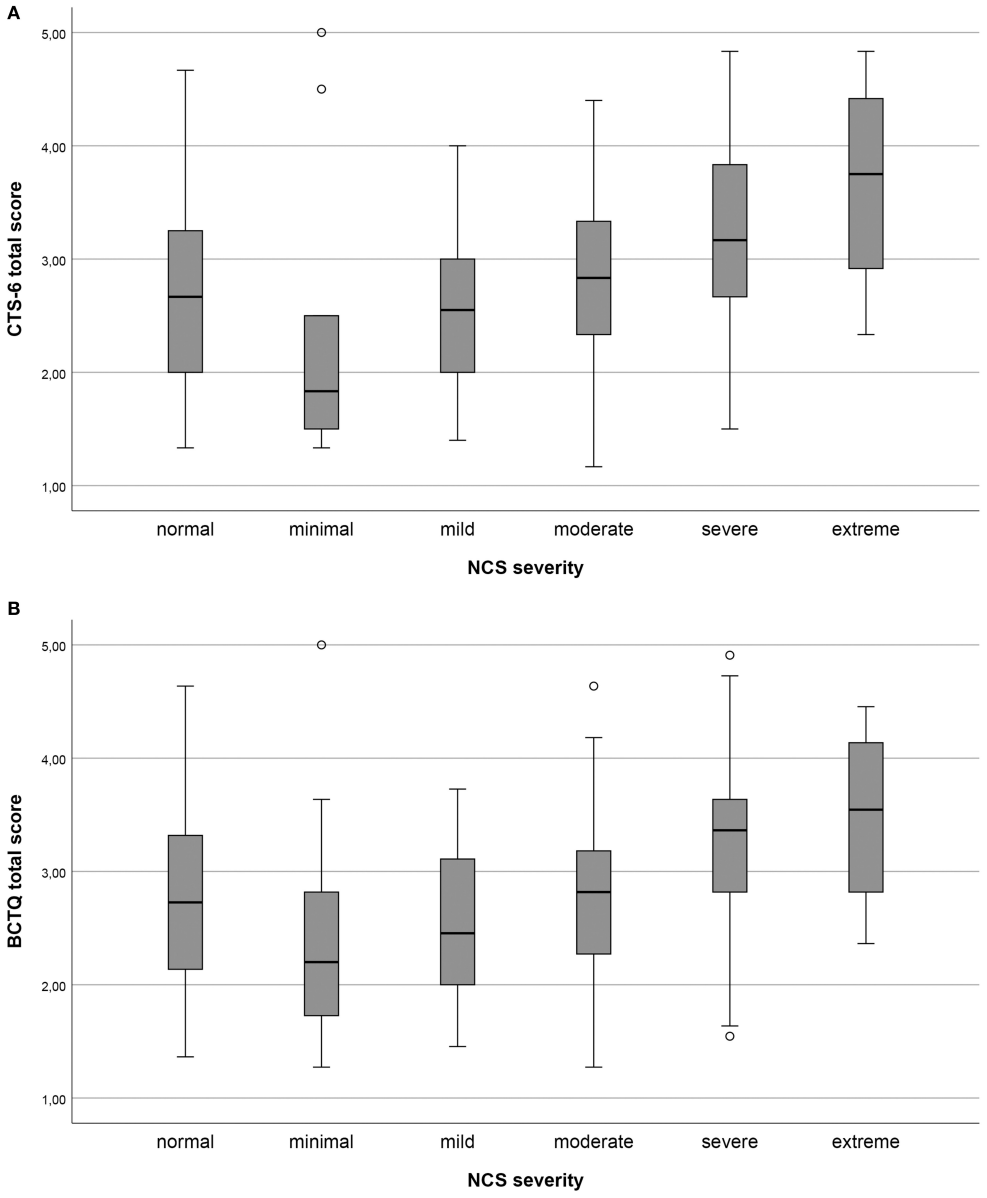
**(A,B)** Total scores of the 6-item CTS (CTS-6) and Boston-CTS (BCTQ), respectively, in the different nerve conduction study (NCS) severity groups of carpal tunnel syndrome (CTS) graded according to Padua. Minimal CTS ≥ 0.5 ms difference between median/ulnar nerves sensory latency; mild CTS, sensory conduction velocities of the median nerve below the lower normal limit; moderate CTS, motor distal latency above the normal limit in addition to sensory conduction velocities of the median nerve below lower normal limit; severe CTS, absent sensory amplitudes in addition to motor distal latency above the normal limit; and extreme CTS, absence of sensory and motor responses.

All pre-defined hypotheses for the correlation between the CTS-6, the BCTQ and the external criteria were confirmed (Table 4). As expected, the total CTS-6 and BCTQ scores (Figures 1A,B) were significantly different between NCS severity groups.

**Table 4:** Construct validity by testing pre-defined hypotheses for the correlation between the 6-item CTS, the Boston Carpal Tunnel Questionnaire and the external measure.

**Hypothesis**	Correlation-coefficient^†^/ *p*-value^*^ found	**Hypothesis confirmed?**
1. In the subgroup of patients with confirmed CTS, we expected a moderate level of correlation between their NCS severity and the total scores in the CTS-6 and the BCTQ.	0.43^†^ (both)	Yes
2. In the whole sample (comprised of patients with and without CTS), we expected a significant, but weak, correlation between the total scores of the CTS-6 and BCTQ and severity of NCS.	0.16^†^ (both)	Yes
3. We expected a significant difference between the NCS severity groups in terms of the severity of pain in the CTS-6 and BCTQ (item #1 in the CTS-6 and item #1 in the BCTQ).	*p* = 0.04^*^; (CTS-6); <0.00^*^ (BCTQ)	Yes
4. We expected a significant difference between the NCS severity groups in terms of the severity of numbness in the CTS-6 and BCTQ (item #5 in the CTS-6 and item #9 in the BCTQ).	*p* = 0.01^*^ (CTS-6); 0.02^*^ (BCTQ)	Yes
5. We expected to find a significant difference between the NCS severity groups in terms of their total CTS-6 and BCTQ scores.	*p* < 0.00^*^ (both)	Yes
6. In patients with confirmed CTS, we expected a high level of correlation between the CTS-6 and the BCTQ scores.	0.8^†^	Yes
7. In patients with confirmed CTS, we expected a low level of correlation between the hand-diagram score and the CTS-6 and BCTQ total scores.	−0.04^†^ (CTS-6); −0.05^†^ (BCTQ)	Yes
8. In the whole sample, we expected a low level of correlation between the hand-diagram score and the CTS-6 and BCTQ scores.	0.01^†^ (CTS-6); 0.01^†^ (BCTQ)	Yes

† *Spearman's rho*;

* *ANOVA*.

### Discriminative Ability

Table 5 shows the results of the discriminative ability testing. Using a score of “probable” as the cut-off for CTS, the hand-diagram showed a high specificity and positive likelihood ratio and good sensitivity in detecting CTS. For the CTS-6 and BCTQ scores of 2.55 and 2.47, respectively, yielded the highest combination of sensitivity and specificity for detecting moderate to severe CTS. Both showed an acceptable to good ability to discriminate between severity levels of CTS.

**Table 5:** Discriminative ability of the 6-item CTS (CTS-6) and the Boston Carpal Tunnel Questionnaire (BCTQ) for detection of moderate to severe CTS in patients with confirmed CTS and of the hand-diagram for detection of CTS in the whole sample.

	**Detection of CTS**	**Detection of moderate-severe CTS**	
	**Hand-diagram**	**CTS-6**	**BCTQ**
Range	Unlikely- classic	1–5	1–5
AUC [95% CI]	NA	0.75 [0.64–0.85]	0.73 [0.62–0.83]
Optimal score	“Probable”	2.55	2.47
Sensitivity [95% CI]	0.72 [0.63–0.79]	0.68 [0.58–0.77]	0.76 [0.66–0.83]
Specificity [95% CI]	0.90 [0.83–0.95]	0.64 [0.42–0.82]	0.64 [0.46–0.83]
Positive likelihood ratio [95% CI]	7.59 [4.28–13.46)]	1.91 [1.11–3.27]	2.11 [1.32–3.93]
Negative likelihood ratio [95% CI]	0.31 [0.23–0.41]	0.50 [0.32–0.74]	0.37 [0.23–0.56]

*NA, not assessed; AUC, area under the curve; optimal score, score with highest sensitivity and specificity combined*.

## Discussion

The Norwegian versions of the CTS-6, BCTQ and hand-diagram showed good measurement properties when assessed in a sample of patients referred for diagnostic work-up for CTS with NCS. The results support the utility of the CTS-6 and BCTQ for symptom severity assessment and of the hand-diagram for diagnostic screening.

The age and gender distribution, as well as the distribution of NCS severity levels, in the present study correspond to previous reports (5, 23, 33). The response rate was high and there were generally few missing items, corresponding to previous studies (29, 34). We did not find floor or ceiling effects in the total scores. However, we found end effects for items concerning numbness and tingling in the CTS-6 and BCTQ, especially in patients with moderate to severe NCS findings (see Figure 1). This group is often considered for surgery (3, 4). End effects can indicate that single items are not differentiated enough, which may make it difficult to measure changes in these items. In turn, this might reduce the utility of the two outcome measures for post-operative follow-up (21).

According to the COSMIN criteria, test–retest reliability of the CTS-6 and BCTQ was very good for both absolute and relative reliability measures, comparable (21, 35) to other translated versions of the CTS-6 (9, 36) and BCTQ (34, 37). Internal consistency was very good for the CTS-6 and BCTQ and comparable to the original version and Spanish versions of the CTS-6. An artificially high Cronbach's α can be found in questionnaires with a large number of items, which is unlikely in our study (21).

All pre-defined hypotheses were confirmed, which supports good construct validity of the CTS-6 and BCTQ. We chose to use NCS as external criteria as they are an objective measurement of nerve function and frequently used to diagnose CTS (38). We found a moderate level of correlation between the NCS severity and the total scores of the CTS-6 and BCTQ in the sample with confirmed CTS, corresponding with previous reports (39). In contrast, the correlation between NCS severity and symptom severity in the whole population was significant, but rather low. This result confirms that the two outcome measures are not specific for CTS and is in accordance with the literature (28, 31, 40). In addition, the correlation between NCS severity and clinical symptom severity in CTS varies in different studies (29, 39, 41–43). This variation might be due to the use of different methods in the studies. For instance, the result would be different if correlation to clinical sum scores or to severity of single symptoms was assessed (28, 30). In our study, pain and numbness intensities, as measured by the CTS-6 and BCTQ, differed between the NCS severity grades, in keeping with previous reports (41). These findings support the notion that NCS and clinical severity assessment are complementary (28, 44) and, consequently, indicate that the CTS-6 and BCTQ should only be used in patients with confirmed CTS.

The high level of correlation between the CTS-6 and BCTQ was expected, as the CTS-6 is derived from the BCTQ. Likewise, the low level of correlation between the CTS-6 and the hand-diagram was expected, as the hand-diagram is designed for diagnostic purposes and not symptom severity assessment in contrast to the CTS-6 and BCTQ. This suggests that the BCTQ would not perform well when used for diagnostic purposes (18) when compared to diagnostic measures.

The hand-diagram had a very good ability to distinguish between patients with and without CTS. This ability is likely due to its measurement of how closely pain and numbness follow the median nerve distribution, which is a classic sign of CTS (1, 31, 32, 45). The CTS-6 and BCTQ showed good sensitivity and acceptable specificity for distinguishing moderate to severe CTS from minimal and mild CTS, as previously reported (29). This finding highlights the clinical utility of the CTS-6 and BCTQ in guiding treatment decisions, as patients with moderate to severe NCS findings often benefit from surgery, and patients with milder severity grades may benefit from conservative treatment (4, 46).

A limitation in this study is that we only assessed the symptom-severity subscale of the BCTQ, and not the functional-impairment subscale. We made this decision because the CTS-6 does not contain a functional impairment subscale. The functional-impairment subscale of the BCTQ should be scored independently form the symptom severity scale (47). Also, we did not test the responsiveness of the CTS-6 and BCTQ, i.e., their ability to detect clinically important changes over time. This knowledge would be necessary to assess the interpretability of the two outcome measures and their utility for follow-up analysis. Because the clinical criteria for CTS partially overlapped with single items in the measures, incorporation bias cannot be ruled out. Incorporation bias is what happens when the test which is being evaluated is integrated into the reference standard. In this case, it may have led to an overestimation of sensitivity of the hand-diagram. Incorporation bias is difficult to avoid and is nearly always present in studies evaluating clinical diagnostic methods, nevertheless, its potential effect should be addressed (48). The items in question are central symptoms of CTS and it is hard to diagnose CTS without asking about paresthesia in the median nerve distribution. We tried to minimize the effect of this type of bias by using a reference standard comprised not only of clinical criteria, but of a combination of clinical criteria and NCS findings. Further, we addressed the problem of bilateral disease by applying the analyses to the most symptomatic side. A limitation is that a Martin-Gruber anastomosis might have been present in some individuals in the sample. Presence of Martin–Gruber anastomosis in patients with CTS might lead to confusing NCS findings (49, 50). Due to the anastomosing fibers bypassing the carpal tunnel, the proximal motor latency (measured at the cubital fossa) and the motor conduction velocity of the median nerve in the forearm can be mistakenly interpreted as normal. As the distal motor latency is usually not impacted by a Martin–Gruber anastomosis, a mismatch between pathological distal motor latency and seemingly normal proximal motor latency can be observed (51, 52). The effect of a Martin Gruber anastomosis on the NCS findings in CTS can be subtle and is not always easy to recognize. This could potentially lead to underestimation of pathology in the median nerve motor conduction velocity in the forearm and in the proximal motor latency in patients with CTS.

It is important to note that the findings of this study are valid in the context of a population referred to NCS with suspected CTS, which is a somewhat select group. However, from a pragmatic standpoint, this sample represents the population in which the studied instruments are likely to be used. It is, for instance, possible to use the hand-diagram to assess a pre-test probability before performing NCS and adapting the scheduled NCS protocol thereafter.

Some major strengths of the present study are that we examined all quality criteria proposed by COSMIN within our design and that we recruited more patients than recommended (21).

## Conclusion

The Norwegian versions of the CTS-6 and BCTQ as well as the hand-diagram showed acceptable to good measurement properties when applied to patients referred to NCS. The hand-diagram provided a clear estimate of pretest probability prior to performing NCS, and the NCS protocol may be adjusted accordingly (53). The CTS-6 and BCTQ provided complementary information to NCS in severity assessment and can be used to guide the therapeutic approach in patients with diagnosed CTS.

## Data Availability Statement

The datasets presented in this article are not readily available because of local data protection restrictions. Requests to access the anonymized datasets should be directed to Daniel Gregor Schulze (d.g.schulze@studmed.uio.no).

## Ethics Statement

The studies involving human participants were reviewed and approved by local data protection official of Oslo university hospital (PVO 2015/14753). The patients/participants provided their written informed consent to participate in this study.

## Author Contributions

DS: data analysis and first draft of the manuscript. DS, KN, JZ, RK, and MG: conception and design of the study, data collection, analysis, and manuscript revision. All authors read and approved the submitted version.

## Conflict of Interest

The authors declare that the research was conducted in the absence of any commercial or financial relationships that could be construed as a potential conflict of interest.

## Publisher's Note

All claims expressed in this article are solely those of the authors and do not necessarily represent those of their affiliated organizations, or those of the publisher, the editors and the reviewers. Any product that may be evaluated in this article, or claim that may be made by its manufacturer, is not guaranteed or endorsed by the publisher.

## Abbreviations

CTS: carpal tunnel syndrome
NCS: nerve conduction studies
AUC: area under the curve
ROC: receiver operating characteristics.
