# Supplementary material for: Clinical Utility of the 6-Item CTS, Boston-CTS, and Hand-Diagram for Carpal Tunnel Syndrome
Source: Front Neurol. 2021 Jul 27;12:683807. doi: 10.3389/fneur.2021.683807 (PMC8353366; doi:10.3389/fneur.2021.683807)
Supplement: Supplementary file 1 [file Table_1.DOC]

| **6-item CTS** |
| --- |
|  |

Angi den hånden som er verst

| Svar på følgende spørsmål når det gjelder |  | Høyre hånd |
| --- | --- | --- |
|  |  | Venstre hånd |

Spørsmålene refererer til dine håndsymptomer en vanlig 24 timers periode i løpet av de to siste ukene (kryss av for ett svaralternativ for hvert spørsmål)

| Hvor sterke er følgende symptomer i hånden? | Ingen | Lite | Moderat | Sterk | Veldig sterk |
| --- | --- | --- | --- | --- | --- |
| 1. Smerte om natten |  |  |  |  |  |
| 2. Smerte om dagen |  |  |  |  |  |
| 3. Nummenhet eller prikninger om natten |  |  |  |  |  |
| 4. Nummenhet eller prikninger om dagen |  |  |  |  |  |

| Hvor ofte våkner du om natten på grunn av følgende symptomer i hånden? | Aldri | En gang | 2 eller 3 ganger | 4 eller 5 ganger | Mer enn 5 ganger |
| --- | --- | --- | --- | --- | --- |
| 5. Smerte |  |  |  |  |  |
| 6. Nummenhet eller prikninger om natten |  |  |  |  |  |

# Atroshi CTS-6 etter Atroshi I et al. The six-item CTS symptoms scale and palmar pain scale in carpal tunnel syndrome. J Hand Surg Am, 2011. 36(5): p. 788-94. Oversatt og validert av Schulze DG, Grotle M, Nilsen KB og Munk R 2015-2018, OUS/HiOA.

Side 1 av 1
