# Supplementary material for: Clinical Utility of the 6-Item CTS, Boston-CTS, and Hand-Diagram for Carpal Tunnel Syndrome
Source: Front Neurol. 2021 Jul 27;12:683807. doi: 10.3389/fneur.2021.683807 (PMC8353366; doi:10.3389/fneur.2021.683807)
Supplement: Supplementary file 2 [file Table_2.doc]

| **Boston carpal tunnel questionnaire**  Skala for grad av symptomer |
| --- |
|  |

Følgende spørsmål refererer til dine symptomer når det gjelder et typisk døgn i løpet av de siste to ukene (marker et svar på hvert spørsmål)

Hvor uttalte er smertene du har i hånden eller håndleddet om natten?

1. Jeg har ikke smerter i hånden eller håndleddet om natten
2. Beskjedne smerter
3. Moderate smerter
4. Sterke smerter
5. Svært sterke smerter

Hvor ofte har smerter i hånd eller håndledd gjort at du har våknet i løpet av en typisk natt de siste to ukene?

1. Aldri
2. En gang
3. To eller tre ganger
4. Fire eller fem ganger
5. Mer enn fem ganger

Pleier du å ha smerter i hånden eller håndleddet på dagtid?

1. Jeg har aldri smerter på dagtid
2. Jeg har beskjedne smerter på dagtid
3. Jeg har moderate smerter på dagtid
4. Jeg har sterke smerter på dagtid
5. Jeg har svært sterke smerter på dagtid

Hvor ofte har du smerter i hånden eller håndleddet på dagtid?

1. Aldri
2. En eller to ganger daglig
3. Tre eller fire ganger daglig
4. Mer enn fem ganger daglig
5. Konstante smerter

I gjennomsnitt, hvor lenge varer en smerteepisode på dagtid?

1. Jeg har aldri smerter på dagtid
2. Mindre enn 10 minutter
3. 10-60 minutter
4. Mer enn 60 minutter
5. Jeg har konstante smerter hele dagen

Opplever du nummenhet (tap av følelse) i hånden?

1. Nei
2. Jeg har beskjeden nummenhet
3. Jeg har moderat nummenhet
4. Jeg har uttalt nummenhet
5. Jeg har svært uttalt nummenhet

Er du svak i hånden eller håndleddet?

1. Ingen svakhet
2. Beskjeden svakhet
3. Moderat svakhet
4. Uttalt svakhet
5. Svært uttalt svakhet

Har du prikkinger i hånden?

1. Ingen prikking
2. Beskjeden prikking
3. Moderat prikking
4. Uttalt prikking
5. Svært uttalt prikking

Hvor uttalt er nummenheten (tap av følelse) eller prikkingen om natten?

1. Jeg har ingen nummenhet eller prikking om natten
2. Beskjeden
3. Moderat
4. Uttalt
5. Svært uttalt

Hvor ofte har nummenhet eller prikking fra hånden vekket deg en typisk natt i løpet av de to siste ukene

1. Aldri
2. En gang
3. To eller tre ganger
4. Fire eller fem ganger
5. Mer enn fem ganger

Har du problemer med å gripe og bruke små gjenstander som nøkler eller kulepenner?

1. Ingen problemer
2. Noe problem
3. Moderate problemer
4. Store problemer
5. Svært store problemer

# Carpal Tunnel Syndrome etter Levine DW et al. A self-Administered Questionnaire for the assesment of severity of symptoms and functional status in Carpal Tunnel Syndrome. [**J Bone Joint Surg Am.**](http://www.ncbi.nlm.nih.gov/pubmed/8245050)1993 Nov;75(11):1585-92. Oversatt og validert av Schulze DG, Grotle M, Nilsen KB og Munk R 2015-2018, OUS/HiOA.
